# Engineered Mitochondrial Transplantation as An Anti-Aging Therapy

**DOI:** 10.14336/AD.2024.0231

**Published:** 2025-05-24

**Authors:** Rui Zhao, Chen Dong, Qian Liang, Jianlin Gao, Chi Sun, Zhifeng Gu, Yujuan Zhu

**Affiliations:** ^1^Research Center of Clinical Medicine, Affiliated Hospital of Nantong University, Nantong, China.; ^2^Research Center of Gerontology and Longevity, Affiliated Hospital of Nantong University, Nantong, China.; ^3^Department of Rheumatology, Research Center of Immunology, Affiliated Hospital of Nantong University, Nantong University, Nantong, China.; ^4^Department of Geriatrics, Affiliated Hospital of Nantong University, Nantong, China.

**Keywords:** Mitochondrial transplantation, Mitochondrial transfer, Anti-aging, Engineered mitochondria

## Abstract

Aging is an inevitable and complex biological process involving multi-factorial mechanisms. Mitochondrial dysfunction is a critical factor in the aging process, characterized by a decline in mitochondrial quality and activity, leading to aging and aging-related diseases. Therefore, mitochondria have become an attractive target in anti-aging therapies. Several senolytic drugs targeting mitochondria and antioxidant agents have been used in anti-aging research in the past few years. However, these strategies may cause adverse effects with long-term medication. In this extensive review, we propose "mitochondrial transplantation," which transfers healthy mitochondria from donor cells to recipient cells to replace damaged or dysfunctional mitochondria, as a new alternative strategy for treating mitochondrial dysfunction and aging-associated diseases. In this review, we introduce the contemporary landscape of mitochondrial transplantation, then discuss intensely the successful applications of mitochondrial transplantation therapy in aging diseases such as neurodegenerative diseases, cardiovascular aging, and reproductive aging, highlighting its translational potential. Finally, we summarize and prospect the challenges and opportunities mitochondrial transplantation faces in anti-aging therapy.

## 1. Introduction

Aging is a slow, gradual, and complex process characterized by changes in tissue and cell functions and structure [1]. With increasing age, many irreversible and degenerative changes occur in the body, such as declined memory, delayed reaction, decreased mobility, reduced hormone secretion, and other external manifestations (e.g., amyloid plaques, skin fold, thinning, and hair whitening). More and more evidence indicate that age increases the risk of chronic diseases, seriously affecting the health of the elderly population and causing social and economic issues [2]. In recent years, life expectancy, as an indicator of population health, has increased worldwide due to medical advances. Prolonging healthy life expectancy and improving quality of life (QoL) have become international priorities. Therefore, investigation on preventive healthcare and anti-aging has become a significant focus of scientific and public health research.

In recent years, with the rapid development in genetics, molecular biology, and cell biology, there has been a deeper insight into the molecular mechanisms of aging. Many studies have proposed that aging varies between individuals as well as between organs within an individual, and a person's biological age is related to a series of complex biomarkers, unlike chronological age [3]. The increase in biological age is typically accompanied by low-grade chronic inflammation and characterized by genomic instability, epigenetic changes, telomere depletion, loss of protein homeostasis, stem cell depletion, considerable autophagy disability, nutrient sensing imbalance, mitochondrial dysfunction, cellular senescence, changes in intercellular communication, and ecological imbalance [4, 5]. Mitochondria are multifunctional organelles crucial in many biological processes, including energy metabolism, signal transduction, and cell fate determination [6]. During the aging process, a decrease in mitochondrial function was observed, which is related to a decrease in oxidative phosphorylation (OXPHOS) activity, cumulation of mutations in mitochondrial DNA (mtDNA), changes in mitochondrial TCA cycle enzyme level, elevated generation of reactive oxygen species (ROS), and disrupted mitochondrial homeostasis [7, 8].

Mitochondrial transplantation involves introducing isolated functional mitochondria into damaged cells or tissues [9]. At present, mitochondrial transplantation therapy, especially engineered mitochondrial transplantation therapy, has been used in age-related diseases with a certain level of safety and effectiveness. In this review, we sum up the current state of mitochondrial transplantation, emphasize the latest progress in its application of anti-aging therapies, and discuss future opportunities, challenges, and perspectives on improving mitochondrial transplantation techniques and accelerating their practical applications.

## 2. Characteristics of mitochondria

### 2.1 Structure of mitochondria

**Figure 1.**
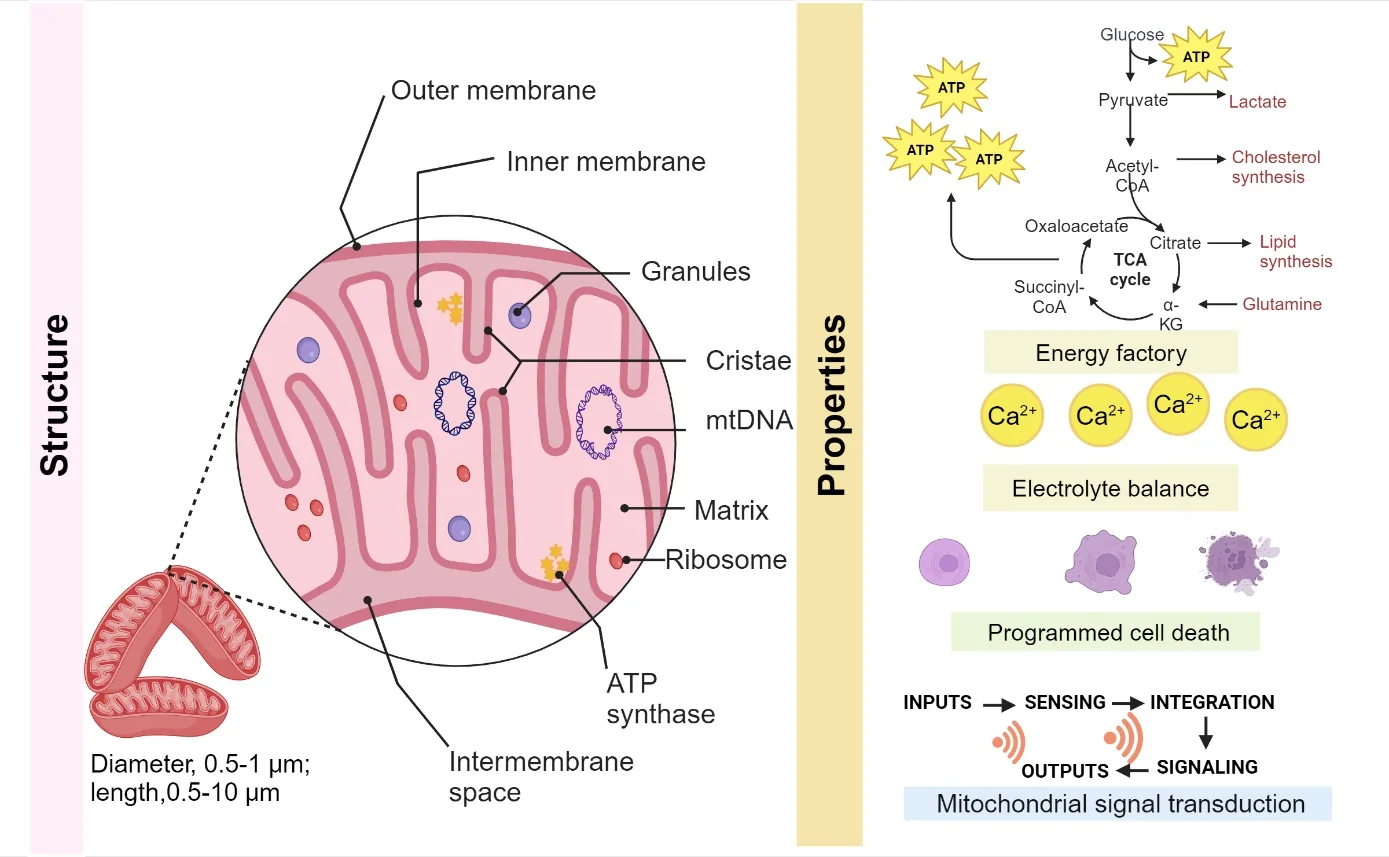
**Mitochondrial structure and properties**. The mitochondrion consists of a double membrane: the inner membrane and the outer membrane, which includes granules, mitochondrial DNA (mtDNA) and ribosomes. Mitochondria are involved in energy supply, electrolyte balance, control of programmed cell death, and mitochondrial signal transduction. The figure was created with BioRender.com.

Mitochondria are double-membrane organelles found in most eukaryotic cells [10]. It is rounded or elongated with a diameter varying between 0.5 μm and 1 μm and a length varying between 0.5 μm to 10-μm (Fig. 1). They may exhibit significant morphological variations among different organisms [11]. There are many mitochondria in the human body, with an average of 300-400 mitochondria per cell. Mitochondria are semiautonomous and semiself-replicating, exhibiting high dynamism. They contain a self-genome, known as mtDNA, located in the mitochondrial matrix, responsible for encoding numerous essential proteins [12, 13]. Mitochondria can synthesize a limited number of mtDNA-encoded proteins, most related to OXPHOS. In addition, the proteins that make up other parts of the mitochondria are encoded by nuclear DNA (nDNA) and synthesized by cytoplasmic ribosomes, which are then transported to their designated functional locations. So, mitochondria are semi-autonomous organelles that function on the nuclear genome.

### 2.2 Properties of mitochondria

Mitochondria, known as the "energy factory", are essential organelles responsible for OXPHOS to produce Adenosine triphosphate (ATP) [14] (Fig. 1). 80% of the energy for cellular life activities is provided by mitochondria [15]. In addition, mitochondria mediate the regulation of various biological processes, such as signal transduction, stress response, intracellular homeostasis, cellular senescence, apoptosis, electrolyte balance, etc. Mitochondria, as the processor of cells, along with the nucleus and other organelles, collectively form a mitochondrial information processing system [16]. Incoming signals are sensed by molecular receptors and biological structures on or within mitochondria. These structures exchange molecular signals and physical states through fusion and fission processes while releasing signaling factors such as metabolites, cofactors, proteins, nucleic acids, and heat, thereby disseminating information beyond the mitochondrial membrane. In addition, mitochondria are also an important node in the intracellular calcium signaling network. Mitochondrial calcium uptake is essential for various cellular functions, such as enhancing ATP production, suppressing autophagy, and controlling cell death [17]. Research has found that mitochondria can cause a "calcium wave" when calcium ions are released, activating second messenger-activated proteins and orchestrating neurotransmitter release [18]. Mitochondria are also involved in calcium ion signaling during cell apoptosis.

### 2.3 Mitochondrial dysfunction and aging

**Figure 2.**
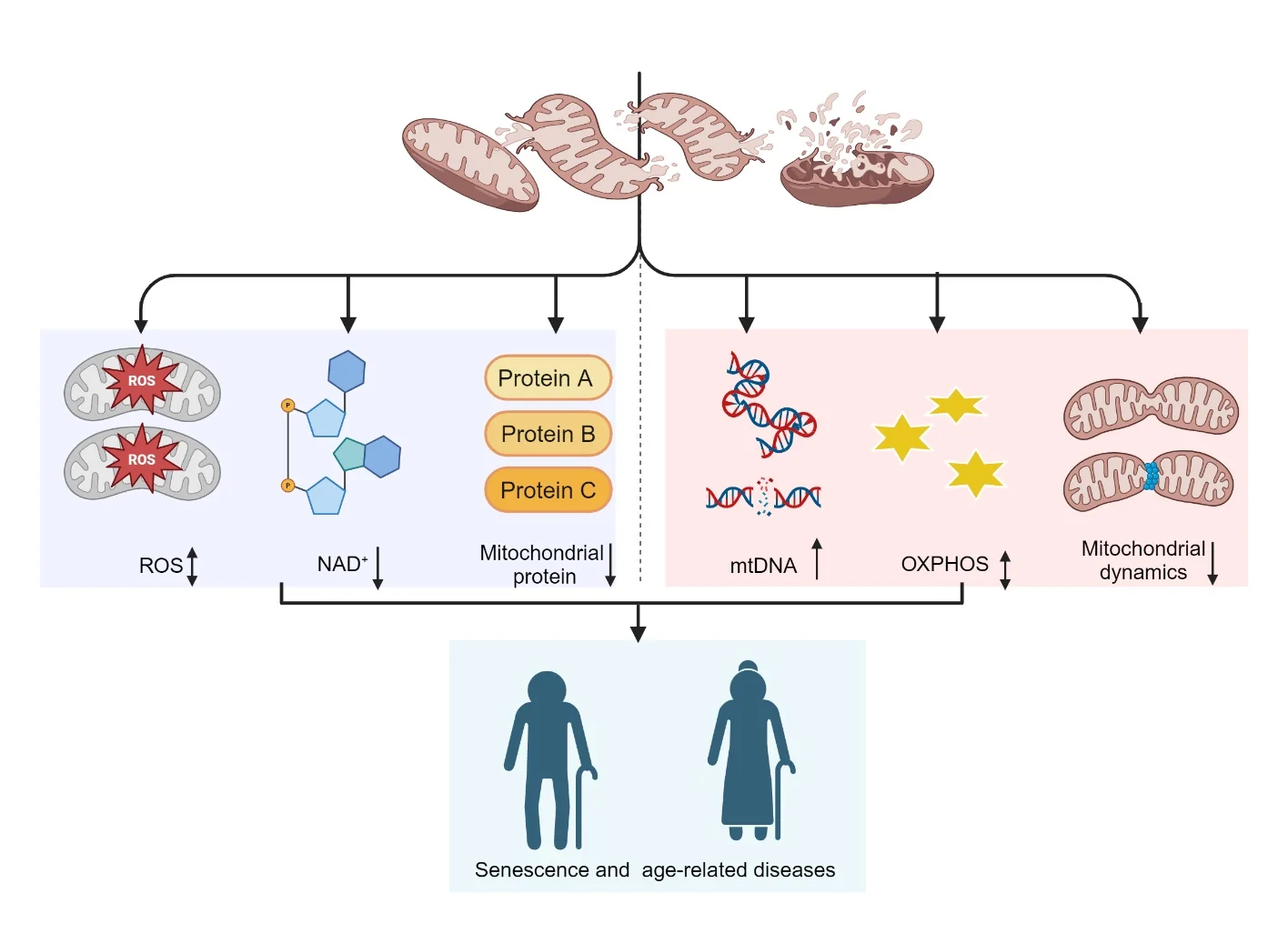
**Characteristics of mitochondrial dysfunction**. The morphological structure of mitochondria changes (such as ridge swelling, fission). Mitochondrial dysfunction is mainly manifested as different levels of reactive oxygen species (ROS), low levels of NAD+, reduced mitochondrial proteins, mitochondrial DNA (mtDNA) damage, reduced mitochondrial dynamics, changes of oxidative phosphorylation (OXPHOS) and so on.

Harman proposed the free radical theory of aging, then redefined by Alexeyev as the mitochondrial theory of aging [19]. The theory suggests that with age, mitochondria, as the main producers of ROS, are a crucial driving factor in aging. In the process of aging and disease, the morphology, quantity, quality control, biogenesis, mitophagy, dynamics, and metabolism of mitochondria change [20] (Fig. 2).

### 2.3.1 morphology and quantity of mitochondria

Relevant studies have shown that the volume and length of mitochondria in aging cells swelled, the number of normal mitochondria decreased, and the number of dysfunctional mitochondria accumulated. There is a phenomenon of mitochondrial division in the structure, with increased fragmentation, cristae discontinuity, decreased matrix density, and the appearance of vacuoles (Fig. 2). Abnormal morphology leads to a decrease in mitochondrial function. Current research indicates that abnormal mitochondria in myocardial cells are involved in myocardial aging [21].

### 2.3.2 mitochondrial quality control

Mitochondrial quality control (MQC) mainly refers to the selective removal of damaged or aging mitochondria and the generation of new mitochondria, which involves mitochondrial biogenesis, mitochondrial dynamics, mitophagy, protein homeostasis, etc [22]. Aging affects the MQC, including protein repair and degradation mechanisms. This can lead to the accumulation and dysfunction of mitochondrial proteins, affecting normal cell function. Research has found that among the Sirtuin family consisting of seven Sirtuins (SIRT1-7), SIRT1-SIRT6 play a crucial role in maintaining mitochondrial quality control, which can improve osteoporosis by regulating mitochondrial protein homeostasis, biogenesis, and mitophagy [23].

### 2.3.2.1 mitochondrial biogenesis

Mitochondrial biogenesis (MB) is a complex process that regulates the number of mitochondria, which is controlled at the transcriptional level by a network of transcription factors and co-regulators [24]. Research has found that aging may affect MB, impairing the synthesis and division of new mitochondria, leading to a decrease in the number and function of mitochondria, and accelerating cellular aging. Numerous studies have shown that a decrease in MB is essential to myocardial injury and heart aging. At the same time, an increase in MB can alleviate mitochondrial dysfunction caused by oxidative stress [25, 26]. Besides, there is evidence that a reduction in MB blunts muscle repair [27].

### 2.3.2.2 mitochondrial dynamics

Mitochondrial dynamics refers to fission, fusion, mitophagy, and transport progress, which are crucial for the optimal function in signal transduction and metabolism [28]. Balanced mitochondrial dynamics provide quality control of mitochondrial networks linked to metabolic status, including biogenesis and turnover and the distribution of mitochondrial DNA [29]. The imbalance of mitochondrial dynamics can disrupt mitochondrial function, leading to abnormal cell fate and a range of diseases, including neurodegenerative diseases, metabolic diseases, cardiovascular diseases, and cancer [30]. Studies have shown that defects in mitochondrial fission/fusion processes have become central regulatory factors of cardiac complications in aging and age-related diseases [31]. The disruption of mitochondrial dynamics can hurt mitochondrial function and myocardial survival, leading to aging-mediated dysfunction and accumulation of mitochondria, directly leading to dilated cardiomyopathy and heart failure. In preclinical models, regulating mitochondrial dynamics with fission inhibitors or fusion promoters can protect mitochondria from pathological damage such as aging. The latest research on C. elegans shows that increased levels of fused mitochondria promote the reconstruction of mitochondrial networks, facilitating longevity [32].

### 2.3.2.3 mitophagy

Mitochondrial dynamics and mitophagy can regulate each other to sustain mitochondrial network homeostasis [28]. Mitochondrial autophagy (mitophagy) plays a vital role in maintaining the quantity and quality of mitochondria by degrading aging or damaged mitochondria through autophagy, which is vital for the energy supply-demand balance in cells and whole organisms, cell differentiation, and developmental programs [33]. Under mitophagy-inducing conditions, mitochondria are labeled with specific molecular markers that recruit autophagy mechanisms to the surface of mitochondria, enclosed into autophagosomes, and transported to lysosomes (vacuoles in yeast) for degradation [34]. Due to damaged mitochondria being the primary source of reactive oxygen species, mitophagy is crucial for mitochondrial quality control and cellular health. Mitophagy defects can lead to the accumulation of damaged mitochondria and impaired cellular function, which may cause pleiotropic pathology, such as excessive inflammation, tissue damage, neurodegeneration, and aging. Inducing mitochondrial autophagy can reduce age-related inflammation and increase the health span [35].

### 2.3.3 Mitochondrial metabolites and related enzymes

Mitochondrial metabolites, such as ROS, Acetyl-CoA, α-ketoglutarate, nicotinamide adenine dinucleotide, as well as other stress signals, can act as signaling molecules to mediate epigenetic modifications and control the expression of metabolic genes. This mechanism is crucial for maintaining cellular stability and regulating cellular aging [36]. Previous studies have shown that during the development of heart failure, mitochondrial energy metabolism homeostasis is disrupted, leading to increased ROS in cardiomyocytes, mitochondrial structural damage, and energy depletion. Emerging studies revealed that mitochondrial-to-nuclear (mito-nuclear) communication through mitochondrial metabolite levels or stress signals changes can lead to various epigenetic changes, promoting efforts to maintain homeostasis and affect aging [7]. The enzymes in mitochondria can be mainly divided into antioxidant enzymes and marker metabolic enzymes. As age increases, the enzyme activity of mitochondria significantly decreases [37]. Research has found that increasing ATP synthase 5 α/β Dimerization can alleviate the senescence of human fibroblasts [38].

## 3. Intercellular Transfer of Mitochondria

**Figure 3.**
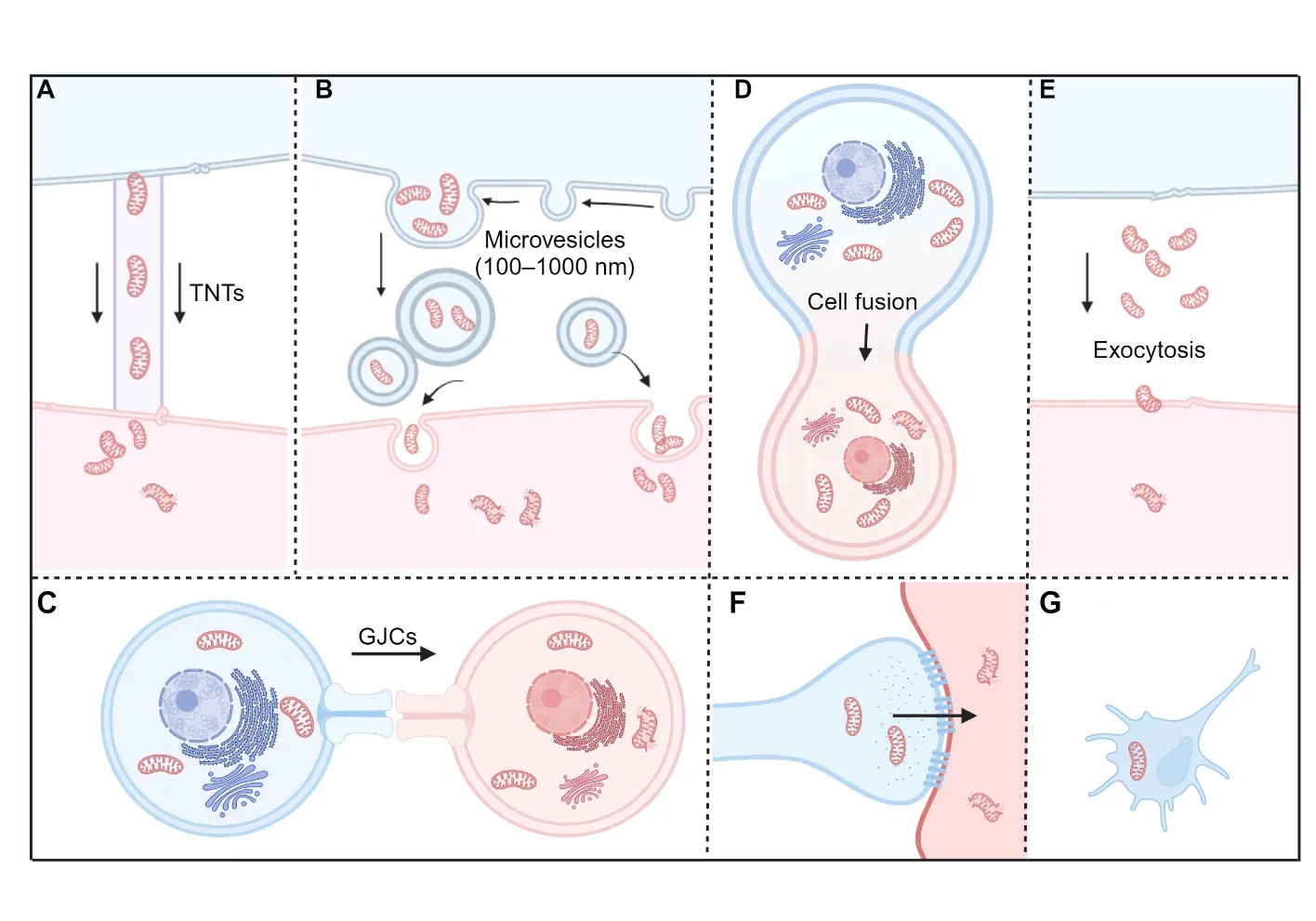
**Intercellular transfer of mitochondria**. (**A**) transfer via tunnelling nanotubes (TNTs); (B) transfer via extracellular vehicles; (C) transfer via gap junction channels (GJCs); (D) transfer via cell fusion; (E) transfer via exocytosis; (F) transfer via synaptosomes; (G) transfer via dendritic networks.

In recent years, the intercellular transport of mitochondria has attracted researchers’ attention and interest. Intercellular transfer of mitochondria can occur through different mechanisms, including tunneling nanotubes (TNTs), extracellular vehicles (EVs), and gap junction channels (GJCs) [39]. In addition, there are some other nonclassical mitochondrial transfers, such as cell fusion [40], synaptosomes [41], and dendritic networks [42](Fig. 3).

### 3.1 Transfer via TNTs

TNT is a linear membranous channel consisting of cell membrane, filamentous actin (F-actin), myosin, and tubulin, first reported by Rustom et al. in 2004 [43]. It is mainly used to mediate intercellular information transmission. F-actin cross-linking within TNTs ensures their structural integrity, providing anti-buckling stability and appropriate protrusion length for its outward growth [44, 45]. In addition, it also facilitates the movement of mitochondria along the cytoskeletal framework of these channels [46]. TNT is the main mitochondrial transfer pattern between cells and can regulate intracellular and extracellular material transport [47]. Its length and diameter vary greatly. This characteristic enables TNT to transfer RNA, proteins, or entire organelles (like endoplasmic reticulum and mitochondria) to adjacent cells or cells located hundreds of micrometers apart [48]. The TNT channel allows for bidirectional and unidirectional material transportation, including various small molecules, organelles, proteins, and virus particles [49-51]. Research has found that mitochondrial transfer is related to physiological states during the formation of the vascular system, such as stress, activation, and stability. Among them, the proportion of cell types, hypoxic stress, and specific factors (such as TNF) can significantly promote the formation of TNT [52]. In summary, as a channel with broad biomedical application potential, TNT is significant in cell survival and physiological functions.

### 3.2 Transfer via EVs

EVs are nanoscale bilayer structured vesicles secreted by cells, which can carry various proteins, lipids, mitochondria, RNA, and miRNA. They participate in mitochondrial transfer to achieve long-distance communication between cells [11, 53, 54]. Based on their structural and biochemical properties, EVs can be categorized into three subtypes [55]. Exosomes are small vesicles (30-150nm in diameter) generated within cells, which merge with the cell membrane and are discharged into the extracellular environment [56]. Microvesicles are tiny vesicles (50-1000nm in diameter) that detach from cell membranes and are released into the cytoplasm mediated through the mediation of grid proteins. Apoptotic bodies are large vesicles (5000nm in diameter) that are the products of programmed cell death. EVs can stably exist in the extracellular fluid and participate in biological processes such as cell proliferation, differentiation, and migration [53, 57, 58]. EVs can transport various types of goods, including various metabolites and mitochondria.

### 3.3 Transfer via GJCs

GJCs are crucial pathways for material and signal exchange among cells, comprising connexons and gap junction proteins (Cx) [59]. They allow the exchange of small molecules such as ions, second messengers, sterols, and phospholipids between cells, playing various physiological functions such as cell communication, providing nutrients, protecting cells, and participating in endocytosis [60-63]. At the same time, GJCs can assist in TNT-mediated mitochondrial transfer [64]. Cx, a transmembrane protein with homologous characteristics, is encoded by various gene families. Currently, more than 20 subtypes of Cx have been identified. Among them, Cx43 stands out as the most extensively researched subtype, involving substance exchange, ion transportation, vesicular transport, mitochondrial respiration, and so on [65]. Gap junctions are vital in cellular physiological processes and have significant implications for regulating cellular function.

### 3.4 Nonclassical mitochondrial transfer

Cell fusion is a process of connecting cells, facilitating material exchange and information transmission between cells. This phenomenon is significant for cell reprogramming and tissue regeneration [66, 67]. Wada et al. [68] research team introduced a method utilizing the Sendai virus envelope to enable the fusion of two independent cells through narrow cytoplasmic connections. In the study conducted by Acquirespace et al. [40], it was observed that when myocardial cells were co-cultured with human pluripotent stem adipocytes, the resulting cell fusion was only partial and not permanent. This partial fusion mechanism facilitates the exchange of substances and mitochondria, thereby promoting the reprogramming of myocardial cells. In addition, studies have shown that mitochondria can be transferred through synaptosomes and dendritic networks [41, 42]. Naked mitochondria can also be released and taken up by cells without a carrier mechanism. This process is referred to as mitochondrial exocytosis (release of mitochondria) and endocytosis (uptake of mitochondria) [69]. Endocytosis of naked mitochondria has been observed in mammalian cells sensitive to chloramphenicol and effapeptin [70].

## 4. Mitochondrial Transplantation

Mitochondrial transplantation/mitochondrial transfer has recently become a potential treatment method in mtDNA-related diseases by transporting healthy mitochondria to damaged cells, tissues, or organs, enabling the recovery of mitochondrial function in diseased cells [15]. Transplantation involves the donor sources of mitochondria and methods of mitochondrial transplantation. This section describes the process of mitochondrial transplantation from the following aspects.

### 4.1 Donor source of mitochondria

**Figure 4.**
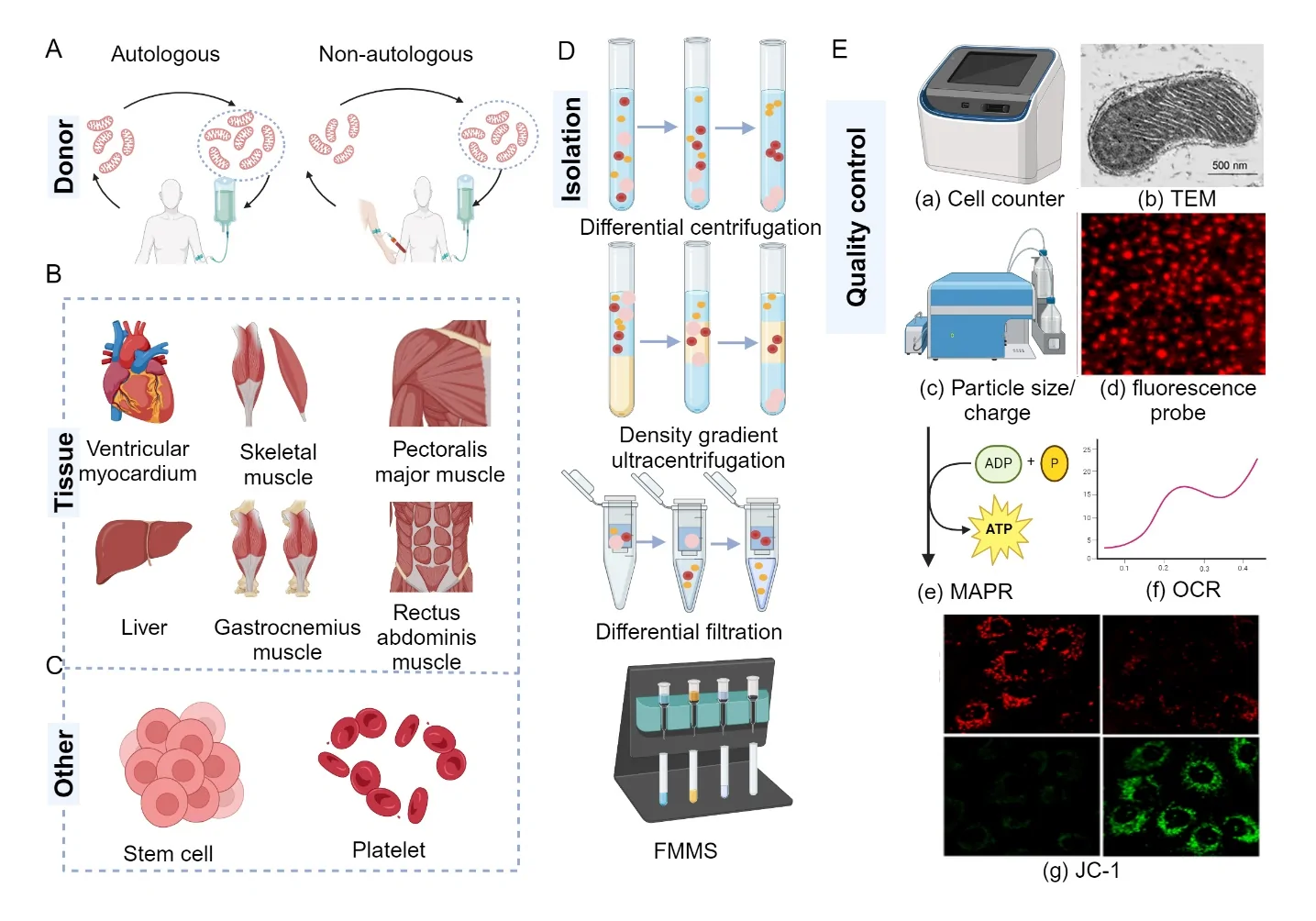
**Mitochondrial transplantation steps**. (**A-C**) donor source of mitochondria; (D) isolation of mitochondria; (E) the quality control of mitochondria. FMMS: fractionated mitochondrial magnetic separation; TEM: transmission electron microscope; MAPR: Mitochondrial ATP produce rate; OCR: oxygen consumption rate. Figure (b/d) is reproduced with permission [97]. Figure (g) comes from instructions (product number: YM0001-3).

From the perspective of donors, mitochondria can be divided into autologous mitochondria and non-autologous mitochondria (Fig. 4A). Autologous mitochondria are an attractive source for mitochondria isolation due to their non- or low immunogenicity, no safety risks, and limited ethical issues. So far, autologous mitochondrial transplantation has been validated in multiple studies to protect the heart from I/R injury [71, 72]. Mitochondria can also be isolated from other people’s healthy tissues, which refers to non-autologous mitochondria. To verify the safety of non-autologous mitochondrial transplantation and determine whether there is an immune or inflammatory response, Ramirez Barbieri et al. [73] and Pour et al. [74] conducted allogeneic and allogeneic/rejection studies. The injection of non-autologous mitochondria did not result in direct or indirect acute or chronic allogeneic reactions or allogeneic recognition reactions, confirming the viability of non-autologous mitochondrial transplantation. Mitochondria can be isolated from almost all normal tissues or cells that are far from the lesion area, for instance, ventricular myocardium [75], skeletal muscle [74], pectoralis major muscle [72], rectus abdominis muscle [76, 77], gastrocnemius muscle [78], Platelets [79], Stem cells [71, 80] and so on (Fig. 4B and 4C). A study comparing the extraction efficiency and quality differences of mitochondria extracted from the hearts, livers, spleens, lungs, kidneys, muscles, and peripheral blood of mice found that the least number of mitochondria were extracted from muscles and peripheral blood [81]. Mitochondria extracted from livers and hearts were successfully extracted with intact structure and high purity. However, mitochondria extracted from livers had lower ATP content and membrane potential than those extracted from hearts.

### 4.2 Isolation of mitochondria

For clinical application, isolation and purification of mitochondria must be timely, with high yield, purity, and activity. The extraction methods of mitochondria include differential centrifugation, density gradient ultracentrifugation, and differential filtration (Fig. 4D). As far back as 1934, Bensley and Hoerr first attempted to isolate mitochondria through cells. Until 1948, well-preserved mitochondria were successfully isolated from rat liver by low-speed centrifugation in salt solution [82]. So far, most methods for isolating mitochondria have relied on differential centrifugation and developed different mitochondrial separation schemes [83]. Differential centrifugation removes intact cells, tissue fragments, and nuclei at low speeds and separates mitochondria from other organelles at high speeds. In addition, due to the decreased density of some abnormal mitochondria, such as swollen mitochondria (e.g., after permeability transition), differential centrifugation can effectively separate intact mitochondria by removing abnormal ones with lower density. However, the efficiency of differential centrifugation is low, and only 0.5 mg of mitochondrial protein can be extracted from 100 mg of muscle tissue [84]. The isolation and purification through differential centrifugation takes a long time, approximately 60-120 minutes to complete, which leads to reduced mitochondrial viability [83].

Density gradient ultracentrifugation uses a linear sucrose gradient (24-54% sucrose) with extremely high centrifugation speed and buoyancy to isolate mitochondria, which is typically used to isolate brain mitochondria with shallow contamination of synaptosomes and myelin sheaths [85-88]. As early as 1990, Sims N.R. et al. [89] used Percoll density gradient centrifugation to isolate metabolically active mitochondria from rat brains and subregions rapidly. The purity of mitochondria isolated by ultracentrifugation is higher [90], while the yield is lower [85] than differential centrifugation. Both methods require repetitive centrifugation steps, decreasing in mitochondrial viability [91, 92].

Fractionated mitochondrial magnetic separation (FMMS) can produce a high-purity, high-yield, and functional mitochondrial solution with the help of magnetic anti-TOM22 antibodies [93] (Fig. 4D). Hubbard WB et al. [85]developed a novel strategy to isolate functional synaptic and non-synaptic mitochondria from mouse cortex and hippocampus by magnetic anti-TOM22 antibodies. FMMS can improve brain-derived mitochondria production for mitochondrial assessment and repair mitochondrial damage in central nervous system (CNS) injuries and neurodegenerative disease.

Most recently, researchers have developed a method to rapidly isolate mitochondria using standardized tissue dissociations and differential filtration [94, 95]. This isolation method replaces the classic differential centrifugation method with differential filtration, avoiding long and repeated centrifugation steps, saving time, and reducing the damage caused by long-term centrifugation. It is simple and fast, can generally separate high-purity, active, and respiratory solid mitochondria [95] within 30 minutes, and can be performed simultaneously with surgery, with better clinical practicality [96].

### 4.3 Quality control of mitochondria

Mitochondrial transplantation requires a high quality of mitochondria. The isolation procedure should be completed at 4°C to maintain mitochondrial health. The extracted mitochondria need to be measured by a blood cell counter, transmission electron microscope, mitochondrial fluorescence probe, Clarke-type electrode, and ATP detection kit to determine the number, purity, vitality, and respiratory function of the isolated mitochondria (Fig. 4E). In general, transmission electron microscope (TEM) is usually used to evaluate the quality of mitochondria. Mitochondria with intact morphology are tubular and show no evidence of significant swelling [97]. However, TEM's analysis of mitochondrial structure and purity is time-consuming, making it unsuitable for rapid evaluation of mitochondrial quality. Mitochondrial function assessment includes measurement of MMP, oxygen consumption, and ATP content. Fluorescence microscopy uses MMP fluorescence probes (such as TMRE, TMRM, and MitoTracker red) to easily and quickly evaluate the mitochondria [98]. MitoTracker Green is another mitochondrial fluorescence probe that does not rely on MMP labeling and targets all mitochondria. Viable mitochondria can be identified by combining MitoTracker Green with any of the MMP fluorescent probes mentioned above. MMP can also use ion-selective electrodes to measure the distribution of tetraphenylphosphonium (TPP) [99]. In addition, the oxygen consumption rate (OCR) detected by Clark-type electrodes and the ATP content measured by ATP luminescence can be further used to evaluate coupled respiration or OXPHOS of mitochondria. For clinical applications, good production practices need to ensure that quality aligns with the criteria for the measurements above. In such scenarios, staining with mitochondrial fluorescence probes may be sufficient to quickly verify mitochondrial mass, as the evaluation can be done within 5-10 minutes.

## 4.4 Mitochondrial transplantation approaches

### 4.4.1 In vitro Methods for Mitochondrial transplantation

**Figure 5.**
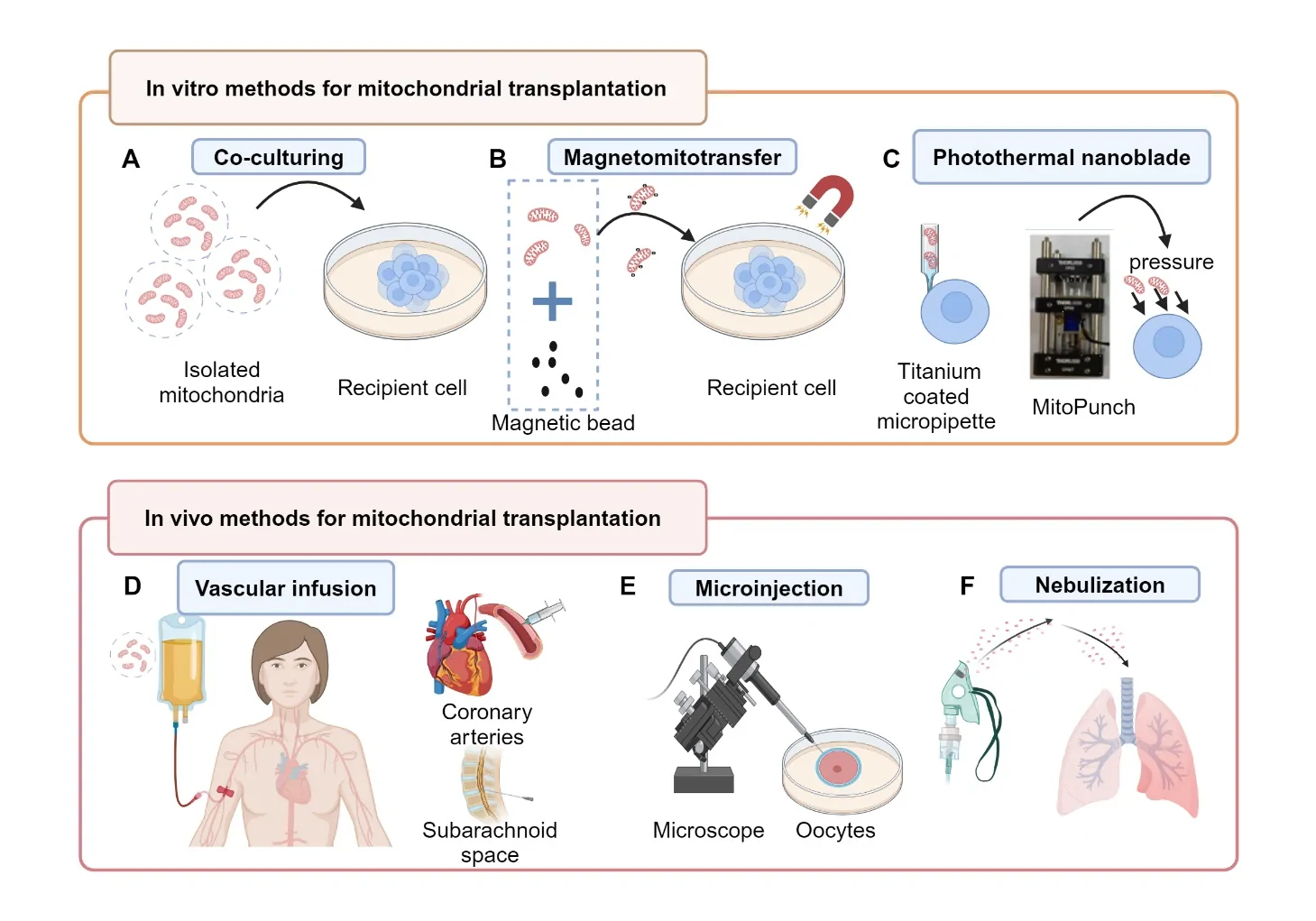
**Mitochondrial transplantation approaches**. Mitochondrial transplantation can be divided into in vivo transplantation and in vitro transplantation. In vitro, mitochondrial transplantation includes co-culturing, magnetomitotransfer, photothermal nanoblade, etc. In vivo, mitochondrial transplantation includes vascular infusion, microinjection, nebulization inhalation, local injection, etc.

In vitro mitochondrial transplantation includes co-culturing, magnetomitotransfer, photothermal nanoblade, etc. (Fig. 5). The co-culturing method refers to the spontaneous transfer of mitochondria into cells by co-culturing purified mitochondria with target cells (Fig. 5A). The transfer of mitochondria in co-cultured cells is dose dependent. The co-cultured mitochondrial transplantation technique is easy to operate, does not alter the extracellular microenvironment, and produces cytotoxic substances, which display advantages in safety and stability. However, due to the limitation of endocytosis, the efficiency of mitochondrial delivery is limited.

Various methods have been used to enhance the internalization efficiency of mitochondria, including synthetic liposomes or cell-penetrating peptide Pep-1 [100], and incorporating additional centrifugation and thermal shock steps through the incubation process [101]. Recently, a technique was developed that utilizes magnetic beads to bind to the mitochondrial outer membrane protein TOM22 and transfer mitochondria with the help of magnetic plates [102] (Fig. 5B). This technique was more efficient and rapid, with an internalization efficiency of 78% - 92%, and have a high ratio of magnetomitotransferred cells and high density of transferred mitochondria within the first day of culture [102]. However, TOM22 can also exist in dysfunctional mitochondria, which could be transported into the cells simultaneously.

Photothermal nanoblade is a technology that uses titanium-coated micropipettes to transfer isolated mitochondria into cells. The micropipette can be rapidly heated by laser pulses, bypassing endocytosis, and cell fusion to open the cell membrane [103] (Fig. 5C). The mitochondrial transfer efficiency of this method is 2%, which is higher than that of microinjection (0.2%-0.3%). "MitoPunch," developed by the University of California, is a pressure-driven mitochondrial transfer device that utilizes existing photothermal nano blades and biophoton laser-assisted cell surgery tool technology to simultaneously transport isolated mitochondria into target mammalian cells [104]. Unlike photothermal nanoblades, MitoPunch requires complex laser and optical systems that utilize pressure to push the separated mitochondrial suspension through porous membrane covering cells into the cytosol of recipient cells [105]. This high-throughput mitochondrial transfer is easy to operate. It allows for consistent transfer of many mitochondria isolated from different donor cell types to multiple receptor cell types, even for non-human species. MitoPunch "pipeline" can produce SIMR (Single-Input Multiple-Output Reprogramming) cells with distinct combinations of mtDNA-nDNA, which can be utilized for a wide range of research purposes and applications across various cell types [105]. Overall, developing various technologies, such as photothermal nanoblades and Mitopunch, can facilitate efficient and controllable intercellular mitochondrial transfer in vitro. Nonetheless, those methods have low throughput and high equipment requirements and cannot be used in vivo.

### 4.4.2 In vivo Methods for Mitochondrial Transplantation

In vivo mitochondrial transplantation includes vascular infusion, microinjection, nebulization inhalation, local injection, etc. (Fig. 5). The vascular infusion method transmits mitochondria into the target organ through the circulatory system, with high efficiency and simple operation (Fig. 5D). Vascular infusion can be administered through arteries or veins. In 2009, McCully et al. [75] first extracted mitochondria from the left ventricular tissue of New Zealand white rabbits. Before reperfusion, viable respiration-competent mitochondria were injected into the ischemic zone of the heart. After 30 minutes of myocardial ischemia, infusion of exogenous mitochondria from coronary arteries led to a remarkable improvement in cell viability and contractile function and significantly decreased infarct size. Exogenous mitochondria can be detected in various heart parts, mainly distributed in adjacent areas or within myocardial cells [106]. Compared with other methods, the vascular infusion transplantation method is closer to clinical practice and more suitable for application in coronary artery bypass grafting or interventional therapy. The vascular infusion method has been utilized in several models, such as New Zealand white rabbits and pigs, as well as various cardiovascular diseases (CVDs). It was not until 2017 that this technology was first applied to the human heart [76, 77].

The microinjection method can inject many mitochondria into the target cell at once, effectively improving the transplantation efficiency (Fig. 5E). Currently, the microinjection method is mainly used for mitochondrial transplantation in individual cells, such as oocytes. Mitochondria are transplanted into oocytes through microinjection, which can repair mitochondrial dysfunction,improve oocyte function and enhance the success rate of in vitro fertilization in elderly females [107]. Pinkert et al. [108] isolated and purified mitochondria from Mus spretus liver, and microinjected the purified mitochondria into the fertilized ova obtained from super ovulated female mice. When using microinjection, each cell in the tissue needs to be injected, with high technical and equipment hurdles to overcome, which pose significant clinical difficulties.

Nebulization is the process of transferring mitochondria into the body through nebulization (Fig. 5F). Moskowitzova et al. [109] transplanted mitochondria through aerosol delivery via trachea (nebulization) or through vascular delivery via a pulmonary artery in a murine lung I/R injury model. Lung tissue recovered after the intervention, with exogenous mitochondria detected in lung tissue, significantly reduced inflammatory cell infiltration, tissue congestion and edema, and lung injury. Nebulization is more suitable for treating respiratory system-related diseases.

### 4.5 Engineered mitochondrial transplantation

**Figure 6.**
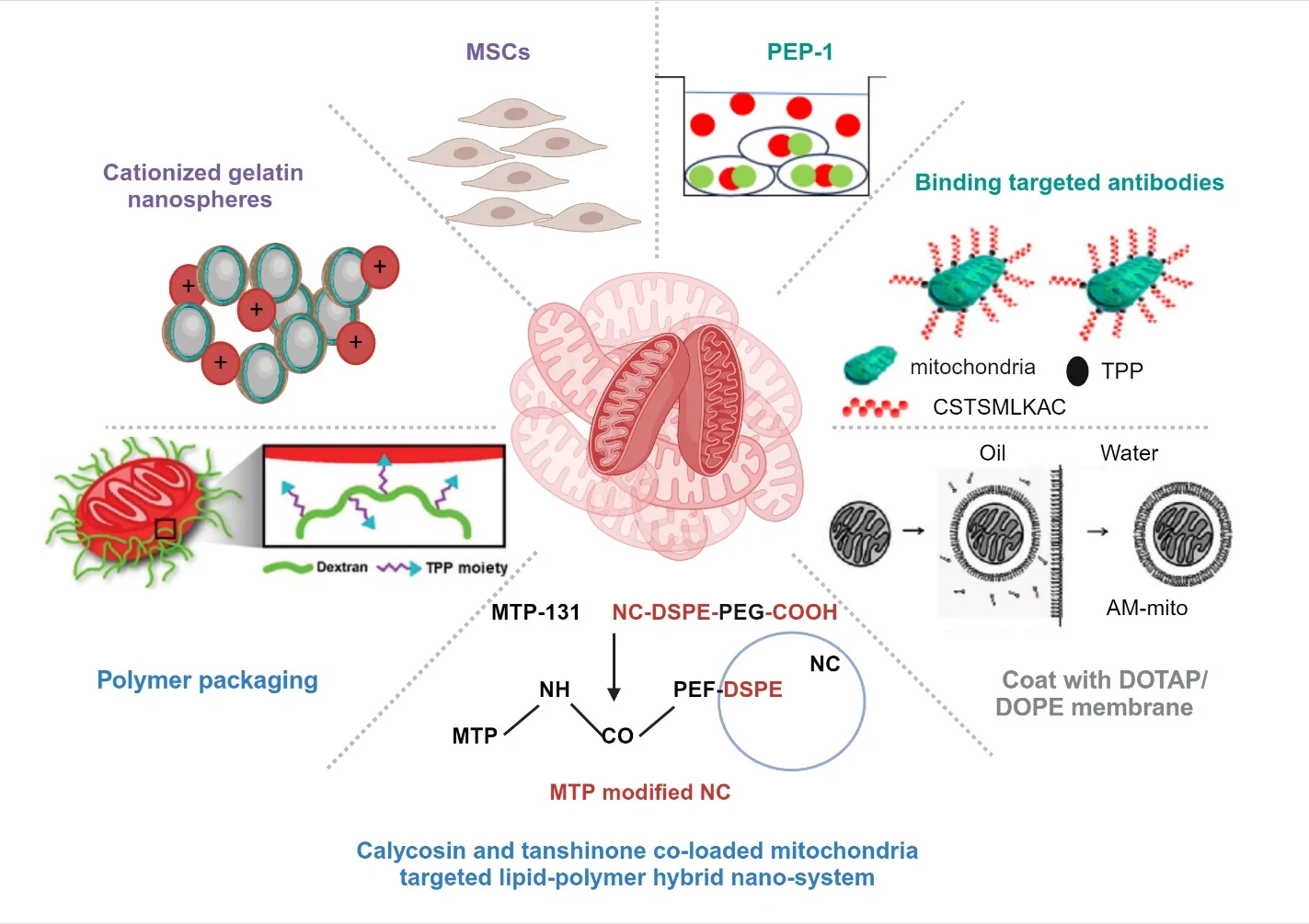
**Mitochondrial modification for transplantation/the existing engineered mitochondrial transplantation and advantage**. Mitochondrial modifications include mesenchymal stem cells, PEP-1, targeted antibodies, cationized gelatin nanospheres, polymer packaging, drugs, and so on. Image reproduced with permission [113, 115, 219]).

Due to electrostatic repulsion between mitochondrial membranes and cells, effectively internalizing mitochondria into intracellular technology is challenging. In order to promote mitochondrial internalization and protect mitochondrial function, in addition to selecting appropriate mitochondrial transplantation methods mentioned above, engineered mitochondrial transplantation is also a popular approach. Engineering mitochondrial transplantation is a type of mitochondrial transplantation that utilizes a set of biomedical techniques to modify mitochondria and optimize the effectiveness of mitochondrial transplantation. Currently, the main techniques for mitochondrial modification include small peptide labeling, liposome transfection, vesicle packaging, and polymer packaging (Fig. 6).

Pep-1 is a cell-penetrating peptide that can bind to mitochondria and promote cell uptake of isolated mitochondria. It does not rely on the endosomal pathway and directly transports isolated mitochondria into the cell through electrostatic and hydrophobic contact with the cell membrane. This may help restore dysfunctional mitochondria without causing them to escape from EVs [110]. Researchers have found that incubating mitochondria with the cell membrane penetrating peptide Pep-1 at 37°C can form a mitochondrial Pup-1 complex. After co-culturing the complex with the recipient cell for 48 hours, it can be observed that over 75% Pep-1 bound mitochondria can be internalized into recipient cells. Chang JC et al. [111] reported that binding with Pep-1 significantly facilitates the maintenance of mitochondrial function and improves therapeutic effects in Parkinson's disease (PD) rats’ models. Compared to conventional co-culture methods, endocytosis effects do not limit this small peptide labeling scheme, and the transfer efficiency can be significantly improved. At the same time, it shortens the mitochondrial exposure time outside the cell, thereby preventing mitochondria from being affected by adverse factors in the extracellular microenvironment [112]. However, the immunogenicity and cytotoxicity of different cell-penetrating peptides must be investigated further.

Mitochondria surface modification can also be achieved using cationic lipids. A study isolated mouse liver mitochondria and labeled them with MitoTracker RedHCMXRos, a probe that binds selectively to the mitochondrial membrane and can be visualized by fluorescence microscopy. Then, the synthetic liposomes, Lipofectin, were used to encapsulate the labeled mitochondria. The successful transfer of mitochondria encapsulated in liposome complexes to cells was observed under fluorescence microscopy, demonstrating that exogenous mitochondria can be transplanted into cells through the liposome synthesis pathway. The coating of artificial 1,2-dioleoyl-3-trimethylammonium propane (DOTAP) and L-α-dioleoyl phosphatidylethanolamine (DOPE) membrane can promote cellular internalization, thereby enhancing neuroprotective effects [113]. It can also transport mitochondria into liposomes or vesicles and play a protective role. There are still some difficulties when many mitochondria need to be transplanted [114].

Functionalizing isolated mitochondria by biocompatible polymers can enhance cell transplantation and, ultimately, in vivo application. Due to its good biocompatibility, dextran has been widely used in a number of biomedical applications. Wu S et al. [115] grafted a polymer conjugate composed of dextran and triphenyl phosphonium onto mitochondria and found that it could protect organelles and facilitate cell internalization. Polymers keep mitochondria in a metabolic sleep state. It can effectively protect mitochondrial respiratory function, promote cell internalization, enhance mitochondrial stability, and extend the retention time of mitochondria in the body. Gelatin, a renowned biocompatible and biodegradable material, comes from collagen and has been widely used in food, medicine, and clinical applications. Yang W et al. [116] used cationized gelatin nanospheres to augment the internalization efficiency of mitochondria.

## 5. Application of Mitochondrial Transplantation in Anti-aging Research

**Figure 7.**
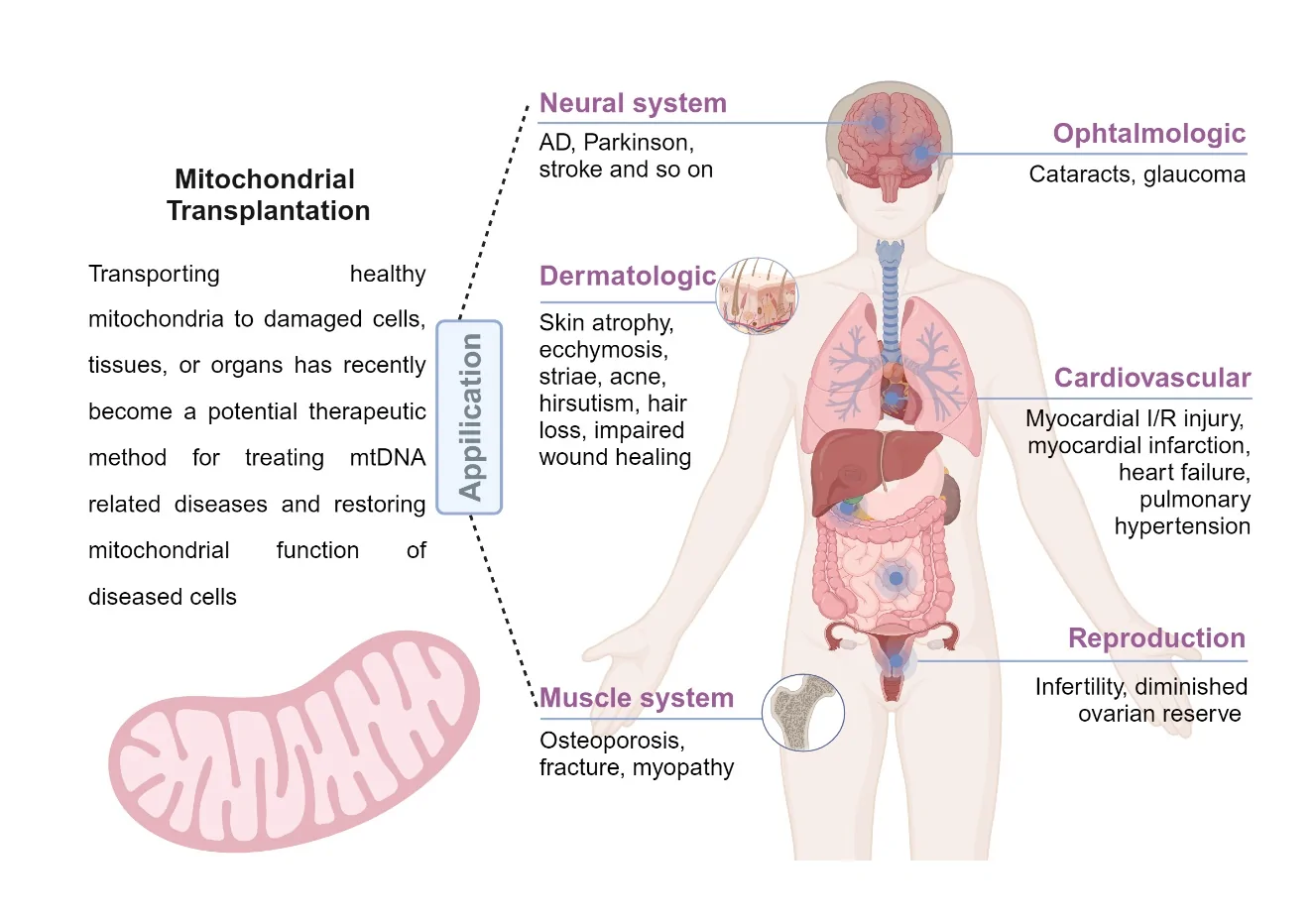
**Application of mitochondrial transplantation in anti-aging research**. The concept of mitochondrial transplantation and its application of mitochondrial transplantation in anti-aging research, such as neural system, dermatologic, cardiovascular, muscle, reproduction systems, and so on. AD: Alzheimer's disease; I/R: Ischemia/Reperfusion.

Mitochondrial disorder is a hallmark of aging-related diseases, including neural degenerative disease, metabolic disorders, and cancer [117]. Mitochondrial transplantation/mitochondrial transfer that transports healthy mitochondria to damaged cells, tissues, or organs has recently become a potential therapeutic method for treating mtDNA-related diseases and restoring the mitochondrial function of diseased cells [117] (Fig. 7). Mitochondria, as an important target for anti-aging, has become a new direction for anti-aging research and drug development. The following aspects will discuss the present progress of mitochondrial transplantation in anti-aging research.

### 5.1 Neural system

As one of the earliest aging organs, the brain undergoes a series of structural and functional changes during physiological aging, such as cerebral atrophy, decreased neurotransmitters, loss of neuronal synapses, and cognitive decline [118]. Under the combined action of certain internal and external factors, the brain gradually develops from physiological aging to pathological aging, ultimately leading to the occurrence of neurodegenerative diseases. Neurodegenerative diseases are a general term for diseases caused by the gradual deterioration of neuronal functions and structures, with notable examples such as Alzheimer's disease (AD), PD, etc [119]. These diseases are age-related, and the incidence rate increases yearly with the increase of the elderly population. The pathogenesis of the above diseases has yet to be fully understood. However, mitochondrial dysfunction and energy metabolism disorder have become a consensus early pathological phenomenon and are increasingly attracting attention [120]. Mitochondrial abnormalities, such as OXPHOS defects, mtDNA defects, accumulation of mitochondrial mutant proteins, and MMP dissipation, play pivotal roles in the progression of early and late-onset neurodegenerative disorders. Concurrently, alterations in mitochondrial structure, including increased mitochondrial breakage and reduced fusion, are closely related to mitochondrial dysfunction and cell death, which are significant in neurodegenerative diseases [121, 122]. As one of the earliest neurodegenerative diseases discovered with mitochondrial dysfunction, PD is characterized by gradual degeneration of dopaminergic neurons and α- Synaptic nuclear proteins enriched in neurons. In PD, various pathogenic processes (such as neuroinflammation) may lead to mitochondrial dysfunction, further resulting in increased oxidative stress, imbalance of bioenergetic, and low survival of damaged dopaminergic neurons in substantia nigra (SN), which ultimately lead to the progression of PD and neurodegeneration [123-126]. Previous studies have reported a significant reduction (30%) in the function of the dense SN, striatum, and complex I activity in the SN in PD brains [127]. AD is a progressive neurodegenerative disease characterized by memory loss, cognitive decline, and behavioral abnormalities. Related studies indicate that mitochondrial dysfunction could lead to numerous pathological changes (such as atrophy and depletion of neurotransmitter systems, loss of synapses and neurons). In the brain and periphery (especially lymphocytes) of AD patients, MMP levels decrease, which leads to elevated levels of oxidative stress and cell apoptosis [128, 129]. Stroke is the main cause of disability and mortality worldwide. Multiple studies have shown that mitochondrial dysfunction (such as the increased production of ROS) causes the occurrence and development of CNS diseases [130]. Timely and effectively improving mitochondrial function is critical to treating CNS diseases.

**Figure 8.**
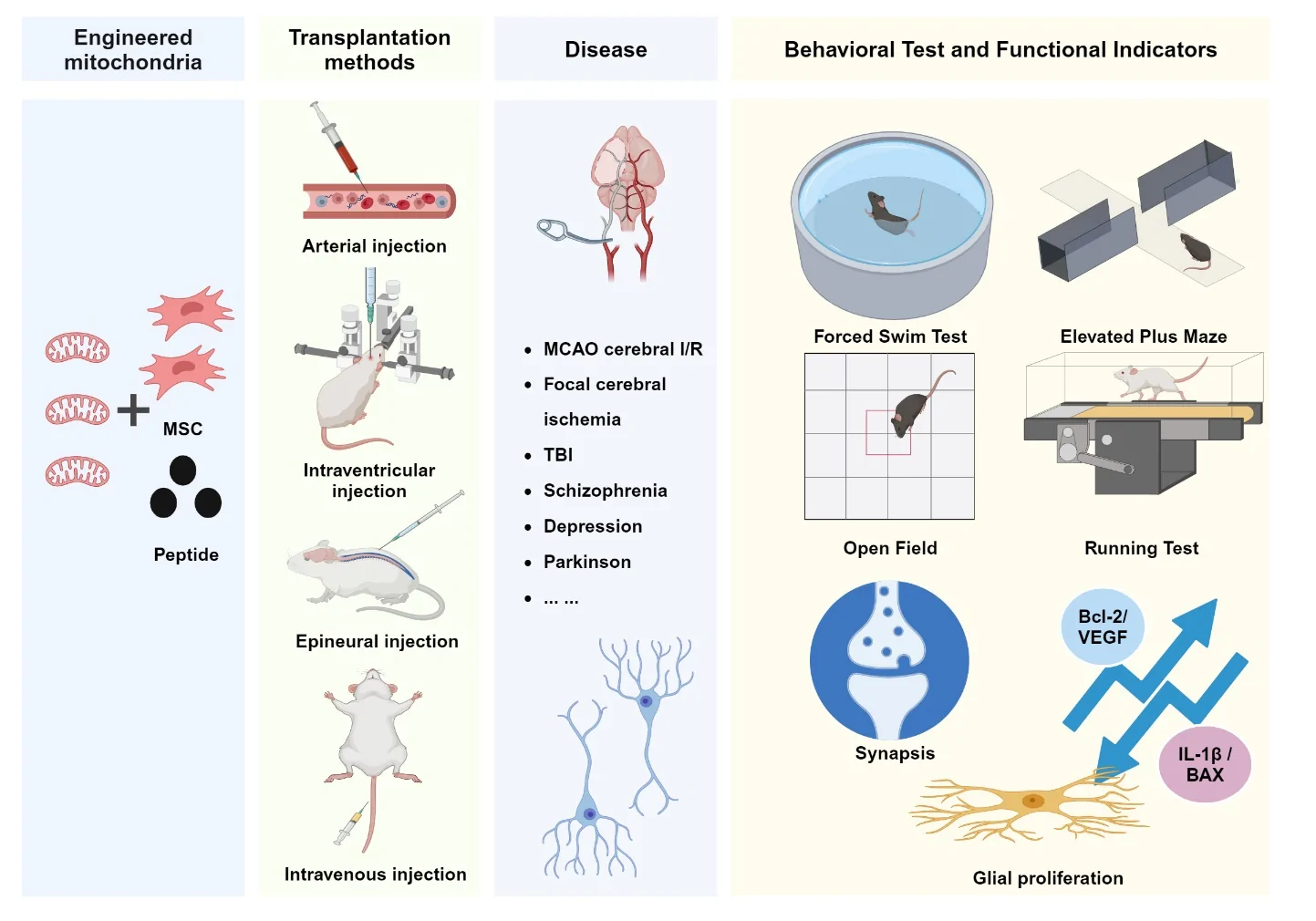
**Mitochondrial transplantation in neurological disorders**. The figure shows mitochondrial transplantation methods, disease types, behavioral tests, and function indicators. MSC: Mesenchymal stem cell; MCAO: middle cerebral artery occlusion; I/R: Ischemia/Reperfusion; TBI: traumatic brain injury.

Healthy mitochondria are crucial for neuronal survival. Given the point of convergence in neurodegeneration caused by mitochondrial abnormalities, antioxidant therapies based on restoring mitochondrial function to repair neuronal damage have received increasing attention. However, traditional antioxidants cannot accumulate specifically in dysfunctional mitochondria and may adversely affect the entire body. Recently, mitochondria-targeted antioxidant compounds, including mitochondrial antioxidants and silencing information regulator 1 (resveratrol), have been thoroughly studied. They can combat oxidative stress and restore neuronal energy supply [119, 131]. These therapies can only alleviate mitochondrial dysfunction but cannot improve mitochondrial dysfunction fundamentally [119]. Mitochondrial transplantation is an innovative and effective neuroprotection strategy [132]. So far, mitochondrial transplantation has positively promoted neurite growth, neuronal viability and activity, benefiting both neural and non-neural disorders [133]. Hayakawa K et al. [134] found that mitochondria can be naturally transferred from healthy astrocytes to improve damaged neurons caused by cerebral ischemia. Wei W et al. [135] found that mesenchymal stem cells (MSCs) combined with therapeutic hypothermia can precipitate better long-term functional outcomes, via increasing mitochondrial transfer, reducing ROS, and increasing neuronal cell viability and ATP. In addition, combination therapy increased Bcl-2 and VEGF and reduced IL-1β and BAX, resulting in a more protective effect on neural function. In neurodegenerative diseases, Chang JC et al. [111] injected peptide-labeled mitochondria into the medial forebrain bundle of the PD rats. A study found that injected mitochondria could resist apoptotic and death oxidative stress, enhance synaptic growth ability, restore the function of dopaminergic neuronal mitochondrial complex I protein in the SN, promote mitochondrial dynamics balance, and ultimately improve movement activity. Similarly, in 6-hydroxydopamine-induced PD rats, mitochondrial transfer therapy based on Gingival MSCs also demonstrates a strong ability to protect neurons and reduce MMP damage and ROS accumulation [136]. Previous studies have found that intravenously injecting healthy mitochondria into AD mice could promote the restoration of the mitochondrial network, reduce hippocampus neuronal loss, increase the activity of citrate synthase and cytochrome c oxidase, promote glial proliferation in the brain area, and significantly enhance the cognitive ability of mice [137]. Up to now, mitochondrial transplantation has been validated in various diseases, such as middle cerebral artery occlusion (MCAO) cerebral I/R [138], focal cerebral ischemia, traumatic brain injury (TBI) [139], schizophrenia [140], depression, diabetic cognitive dysfunction [79], PD, etc [141]. The transplantation methods include arterial injection, intraventricular injection, epineural injection, intravenous injection, vitreous injection, and spinal resection (Fig. 8). Reviewed representative studies on mitochondrial transplantation in nervous system disease over the past five years, Supplementary Table 1 summarizes the experimental details, including the model, dosage, source, and administration route of mitochondria (Supplementary Table 1). It was found that research has only focused on animal-related experiments, the mitochondrial source, times and amounts of injections, and the duration of treatment vary greatly. However, most experiments can improve cognitive impairment by increasing neuronal activity, enhancing mitochondrial metabolism, and reducing neuroinflammation.

### 5.2 Cardiovascular system

Vascular aging is a major risk factor for CVDs and health of the elderly [142]. With increasing age, the elderly experienced endothelial dysfunction, large elastic arteriosclerosis, and other vascular dysfunctions [143]. Mitochondria are important in maintaining vascular homeostasis. Besides the functions in myocardial bioenergetics, it also plays essential roles in redox balance, anabolism and catabolism, as well as the initiation of inflammatory and fatal signals in the cardiovascular system. Age- and disease-related mitochondrial dysfunction led to vascular dysfunction, thereby increasing the risk of CVDs. Professor Guido Kroemer and Professor Mahmoud Abdellatif creatively pointed out that mitochondrial dysfunction is one of the eight molecular hallmarks of cardiovascular aging [144]. It has been confirmed that the mitochondrial function of aging myocardium is damaged at varying degrees, including the changes in mitochondrial content and morphology, the opening of permeability transition pore, expression and activity of electron transport chain complex, formation of ROS, metabolism and dynamics [145]. The mitochondria in an aging heart display increased diameter (swelling), ridge loss, matrix deformation, and reduced OXPHOS secondary to the decreased activity and stability of mitochondrial respiratory chain complexes, particularly complex IV, as well as myocardial cell enlargement and decreased contractility [146].

Given the relationship between mitochondrial dysfunction and vascular aging, it is feasible to consider mitochondria as a target for vascular aging and to restore mitochondrial function in the elderly cardiovascular system through exogenous and fully functional mitochondrial infusion [147]. Numerous pieces of evidence suggest that transplanted healthy mitochondria improve cardiac function and prognosis. Mitochondrial transplantation can confer cardioprotection through mitochondrial abundance and metabolic fitness. The transplanted mitochondria can restore MMP, preferentially restore the contraction function of ventricular cardiomyocytes, boost cell cycle status, inhibit apoptosis, and ultimately improve cardiac function and outcomes [148]. Currently, naked mitochondrial and cell-mediated mitochondrial transplantation are used to treat CVD [149]. Naked mitochondrial transplantation uses microinjection technology to directly inject mitochondria separated from healthy tissues or cells into the myocardium, coronary arteries, or veins to improve cardiac function [150]. Although this technology does not have the hazard of complications like autoimmune responses, intramyocardial hematoma, arrhythmia, and microvascular occlusion, it has high technical demands [151] (Table 1). Cell-mediated mitochondrial transplantation is mainly mediated by TNT, cell fusion and EV. In addition, synaptic complexes and dendritic networks mediated by gap junction channels can mediate mitochondrial transplantation, but it has not been reported in the CVD [149]. Transplantation of engineered mitochondria mediated by MSCs into endothelial cells can enhance aerobic respiration, protect myocardial cells from oxidative stress damage, and inhibit left ventricular dilatation and myocardial fibrosis [152]. In the latest research, Lin et al. [52] devised a strategy to artificially transplant mitochondria, transiently enhancing EC bioenergetics and enabling them to form functional vessels in ischaemic tissues without the support of MSCs. The fusion of MSCs with cardiomyocytes saved damaged cells through a paracrine mechanism. However, the cell fusion transplantation method has many safety issues, such as microcirculation obstruction and arrhythmia, which limit its application [151]. The EV mediated mitochondrial transfer method can be used for various diseases and maintain high mitochondrial stability without the risk of microvascular occlusion and arrhythmia. The origin of mitochondria is a significant issue. Currently, most studies use mitochondria derived from autologous mitochondria. Ibáñez et al. [153] and Brestoff et al. [154] secrete mitochondria from myocardial cells from different tissue sources and transfer them to damaged myocardial cells through EV. Studies have found that mitochondria from different sources can improve I/R injury and myocardial infarction. Up to now, many preclinical trials have validated in animal models that mitochondrial transplantation enables enhanced cardiac function in CVDs, such as I/R injury, myocardial infarction, heart failure, pulmonary hypertension, and ischemic stroke [75]. Moreover, in 2017, Emani, S. M. et al. [76] performed the first clinical application of mitochondrial transplantation, and the study found that pediatric patients suffering from myocardial ischemia-reperfusion injury received good relief after mitochondrial transplantation. The new technology of mitochondrial transplantation has broad prospects in CVDs, even though it is still in its early stages, with many questions remaining unanswered.

**Table 1:** Comparison of mitochondria transplantation methods.

Methods	Safety	Efficacy
**Microinjection [130]**	Relatively safe while high technical demanding	Improve cardiac function
**Co-incubation [129]**	Have safety issues such as microcirculation obstruction and arrhythmia	Enhance aerobic respiration, protect myocardial cells from oxidative stress damage, and inhibit left ventricular dilatation and myocardial fibrosis
**EVs [132]**	Maintain high mitochondrial stability; low risk	Improve I/R injury and myocardial infarction

EVs: extracellular vehicles; I/R: Ischemia/Reperfution

### 5.3 Reproductive system

**Figure 9.**
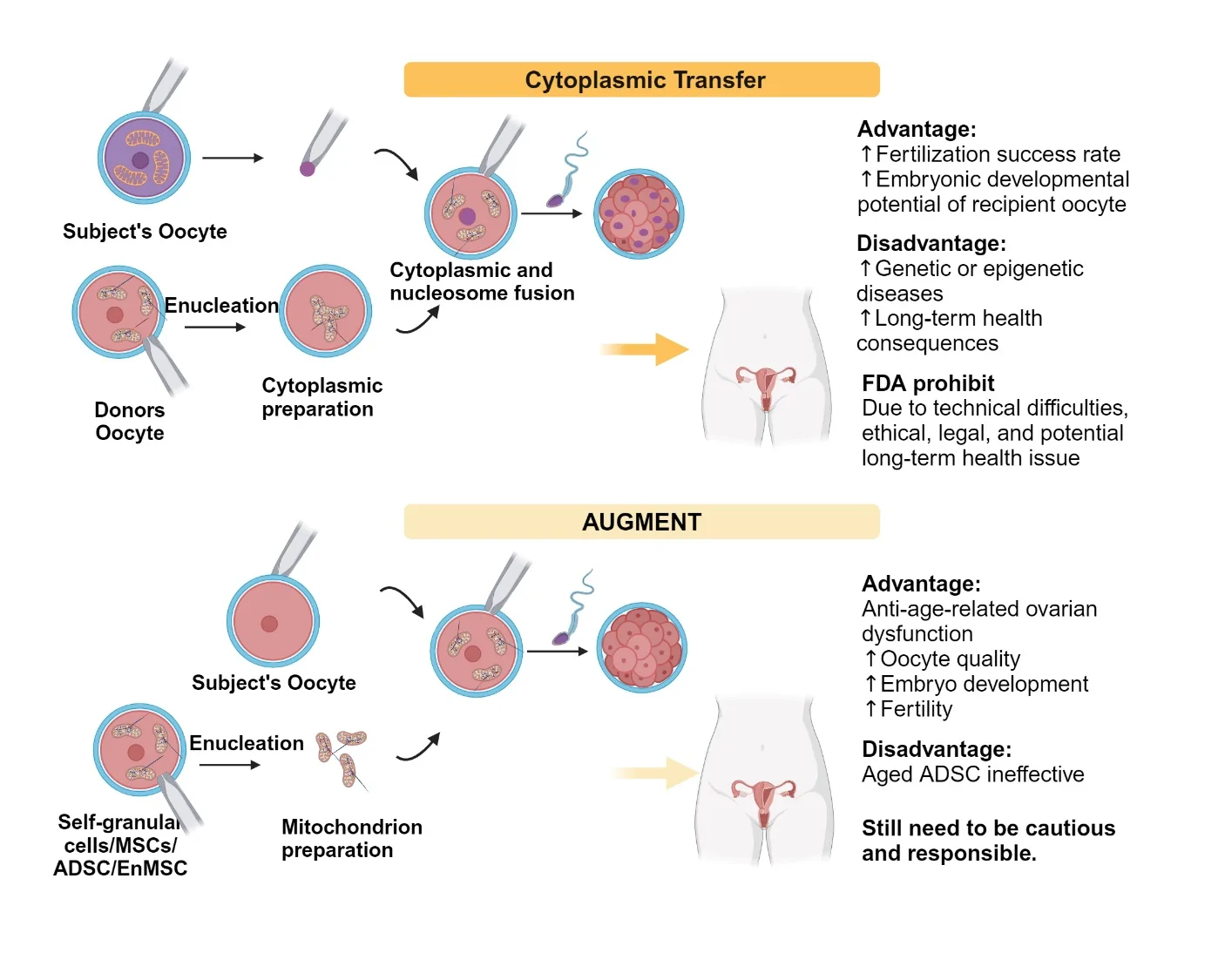
**Mitochondrial transplantation in reproductive system**. The figure shows two methods: cytoplasmic transfer and autologous germline mitochondrial energy transfer (AUGMENT), both of which have certain advantages and disadvantages. FDA: Food and Drug Administration; ADSC: Autologous adipose tissue-derived stem cell; EnMSC: Endometrial mesenchymal stem cell.

Diminished ovarian reserve (DOR) is a dangerous signal of fertility decline, and the clinical incidence rate is increasing year by year, and gradually younger [155]. Research shows that mitochondrial activity has been implicated as a key factor regulating female reproductive processes [156]. Mitochondrial dysfunction, such as a decrease in the amounts of mitochondria, changes in mitochondrial morphology, structure and space, genetic abnormalities in mitochondria, and abnormalities in mitochondrial autophagy can cause dysfunction and apoptosis of oocytes and their surrounding cumulus granulosa cells, leading to follicular atresia and a decrease in ovarian reserve function [157, 158]. Liu et al. [159] observed the proportion of elongated mitochondria with numerous cristae and more high-density matrix particles in elderly females. Mitochondria are often distributed in areas with high energy demand and active metabolism. The irregular distribution of mitochondria is a sign of oocyte damage. Tarazona et al. [160] found that the mitochondrial activity of immature oocytes was deficient, showing diffuse or unclear differentiation. mtDNA is the only carrier for oocyte mitochondria to transfer genetic material to the next generation. Studies have shown that mtDNA copy number variation and structural mutation accumulation can lead to mitochondrial dysfunction and reduced intracellular ATP synthesis, affecting the quality of oocytes and ovarian reserve function. Mitochondrial autophagy is a key regulator of intracellular homeostasis. Song et al. [161] have shown that the targeted deletion of the critical autophagy gene Atg7 in germ cells can lead to reduced fertility in female mice, which further indicates that autophagy-associated genes could potentially serve as pathogenic factors for primary ovarian insufficiency in women. Abnormal morphology and functional destruction of the mitochondria are important mechanisms that cause DOR. Current treatments mainly include drug therapy and lifestyle interventions, such as the use of antioxidants, melatonin, growth hormones, and so on [162]. Recently, with the development of micromanipulation technology, mitochondrial technology has provided a new possibility to treat DOR. Artificial mitochondrial transplantation can replace functional mitochondria in germ cells as a new approach for DOR treatment. By supplementing this substance, damaged mitochondria can be alleviated, the quality of oocytes can be improved, and recovery to a youthful level. At present, there are three main methods for artificial mitochondrial transplantation: cytoplasmic transfer, allogeneic somatic cell mitochondrial transplantation, and autologous germline mitochondrial energy transfer (AUGMENT) [163, 164] (Fig. 9). Cytoplasmic transfer enhances the fertilization success rate and boosts the embryonic developmental potential of recipient eggs [165], but due to the presence of mtDNA, it can lead to mitochondrial heterogeneity in offspring, further leading to genetic or epigenetic diseases [166]. Previous reports pointed out that cytoplasmic transfer can lead to long-term health consequences, such as systemic hypertension and increased weight and fat mass at all ages [167]. Accordingly, heterologous cytoplasmic transfer, although promising, due to technical difficulties, ethical, legal, and potential long-term health problems, the United States Food and Drug Administration (FDA) has closed the clinical protocol of cytoplasmic transfer [168, 169]. There are no ethical issues with AUGMENT, but the source of mitochondria is still controversial. Granular cells, the tissue cells closest to the egg, provide nutrients and regulatory factors during the development of the egg. The mitochondrial morphology and function of the two types of cells have great similarities. Kong Linghong et al. [170] found that self-mitochondria transfer from self-granular cells can enhance the quality of the embryo and increase the pregnancy rate without affecting the fertilization rate. However, aging also affects granulosa cells with accumulation of mitochondrial damage and mtDNA mutations [171]. Not all cells can serve as donors for mitochondrial transplantation. Due to the cellular specificity of mitochondria, mitochondria derived from somatic cells such as liver cells are insufficient for rejuvenation [172]. MSCs, present in various tissues such as the umbilical cord, placenta, and bone marrow, can be induced to differentiate into other types of cells, possessing multidirectional differentiation potential. Using MSCs as the sources of mitochondria to supplement oocytes is a promising technology, as stem cells are similar to mitochondria in MII oocytes, and the harvest and cultivation of MSCs are more convenient and stable compared with oogonial stem cells [173, 174]. Wang Lingjuan et al. [175] found that intra-ovarian injection of MSCs could exert the most significant anti-age-related ovarian dysfunction effects within a month. Autologous adipose tissue-derived stem cell (ADSC) is also utilized as mitochondrial sources to supplement oocytes. Yang Yiet al. [176] and Wang Zhenbo et al. [177] used adipose-derived stem cells from aged mice and found mitochondria from ADSCs can enhance oocyte quality, embryo development and fertility. However, Sheng Xiaoqiang et al. [178] did not find the above advantages in C57BL/6 mice. In 1978, endometrial stem cells (EnMSC) were first proposed, which possess robust regenerative capabilities, higher Ψm, and lower ROS levels [179]. Zhang Qi et al. [180] has found that supplementing mitochondria derived from EnMSCs can enhance the quality of oocytes in aged mice and stimulate embryo development. In summary, the benefits of mitochondrial transplantation are significant, but when applying mitochondrial transplantation technology to assisted reproduction, people need to choose a more cautious and responsible approach.

### 5.4 Muscle system

Skeletal muscles account for approximately 50% of the human body weight and play a crucial role in exercise, heat production, and overall metabolic homeostasis [181]. With the aging process of society, sarcopenia and elderly frailty have attracted widespread attention and have become a hot topic in gerontology research. Muscle deficiency increases the risk of falls, fractures, and mortality in elderly people, affects daily living ability, leads to limited activity and negative impacts on QoL, and places a heavy burden on individuals, society, and the economy. Decreased mitochondrial function in skeletal muscle with age has been widely studied, and many clinical studies have suggested the relationship between mitochondrial abnormalities and skeletal muscle aging. Mitochondrial signaling pathways are involved in skeletal muscle atrophy, such as autophagy and mitophagy, the ubiquitin-proteasome system, mitochondrial biogenesis, and mitochondrial dynamics [182, 183]. Reduced mitochondrial function of skeletal muscles is associated with slower walking speed, lower muscle strength, fatigue, and sarcopenia [184, 185]. In muscle tissue, when mtDNA heterogeneity exceeds a certain level or mitochondrial dysfunction occurs, less ATP and more ROS will be produced, leading to muscle atrophy, weakness, and endurance loss. The study of muscle samples from healthy people found that with age, the number of mitochondria decreased [186], and the activity of Citric acid cycle enzymes, oxygen consumption, and ATP synthesis decreased [187]. Canadian researchers found that the mitochondrial morphology of atrophic skeletal muscle in elderly mice changed. Muscle reduction was related to larger and less circular subsarcolemmal mitochondria, while aging intermyofibrillar mitochondria were longer and more branched, indicating an increase in fusion and/or decrease in fission progress, which can disrupt mitochondrial function and impair mitophagy [188]. In summary, mitochondrial dysfunction is the main mechanism leading to skeletal muscle atrophy; therefore, studying how to restore or improve mitochondrial function to promote muscle regeneration is significant.

Lifestyle changes, such as physical activities, nutrition, and medical interventions, are partially effective in treating sarcopenia but are insufficient to overcome it. Considering the vital role of mitochondria in skeletal muscle function and metabolism, mitochondrial transplantation could be a potential therapeutic approach for improving skeletal muscle health and treating sarcopenia [189]. Despite advancements in mitochondrial transplantation research, its utilization in treating sarcopenia remains somewhat constrained. A study from Canada found that intermyofibrillar murine skeletal muscle mitochondria can restore the functional capacities of the myoblasts, and the incorporation of mitochondria into myoblasts increases in a dose- and time-dependent manner. In addition, muscle mitochondria can also be effectively transferred into human fibroblasts with mtDNA mutations, thereby enhancing mitochondrial dynamics and metabolic functions, rescue and maintaining cellular homeostasis [190]. Research in Korea also shows that after normal mitochondriatransferred into atrophic muscle cells, ATP production, MMP, ROS level, and target cells OCR are restored. The AMPK/FoxO3/Atrogene pathway in atrophic muscle cells is blocked, and muscle cell atrophy is controlled [101]. Although the above methods are simple and easy to use, the current methods of mitochondrial transfer have a low throughput and pose the risk of immune reactions following high-dose injections, which greatly impedes clinical applicability. To overcome these challenges, Professor Sun Dong's team from the City University of Hong Kong has pioneered a droplet microfluidics-based technique for mitochondrial transfer. This innovative approach enables precise and high-throughput mitochondrial delivery to individual cells, leveraging the benefits of coculture and droplet microfluidics. Notably, this technology demonstrates promising therapeutic outcomes in muscle regeneration [191]. Additionally, MSC transplantation therapy may be an effective intervention to alleviate and prevent the development of musculoskeletal degenerative changes. Based on mitochondria transplantation from stem cells for mitigating Sarcopenia, it can achieve dual protective effects of mitochondrial delivery pathway and stem cells [192]. In summary, therapies restoring mitochondrial function are promising treatment strategies for studying the alleviation and treatment of sarcopenia, which can help improve QoL and the lifespan of elderly people. This field is worthy of continuous attention for the development of geriatric medicine.

### 5.5 Integumentary System

The skin is the first line of defense against environmental hazards. Internal mechanisms such as programmed aging and cellular senescence and extrinsic mechanisms such as ultraviolet radiation, smoking, heat and air pollution can jointly lead to skin aging and changes in skin structure and physiology [193, 194]. Skin photoaging is mainly manifested as rough, thickened, dryness, severe loss of elasticity, relatively coarse wrinkles, depigmentation or Telangiectasia in local areas. It can develop into squamous cell carcinoma, solar keratosis, malignant melanoma, and other benign or malignant tumors [195]. The mitochondrial mutation theory suggests accumulated mtDNA mutations as a biomarker of skin aging, especially skin photoaging [196, 197]. mtDNA damage leads to mutation, which hurts the translation of proteins involved in the electron transport chain, resulting in ROS excess and metabolic dysfunction. In addition, mitochondrial ROS increased mtDNA mutations, further accelerating ROS production, ultimately leading to cell aging and apoptosis [198]. Over time, the accumulation of damaged mitochondria can reduce the skin's regenerative capacity, promote structural weakening, disrupt inflammatory response, and increase the risk of cancer [199]. Research has found that mutations in mtDNA encoding the respiratory chain subunits are often detected in the skin of elderly people, especially in areas exposed to sunlight. The disturbance of OXPHOS function caused by mtDNA damage is related to the skin aging phenotype [200-202]. The mitochondrial network in aged Keratinocytes showed a fragmented phenotype and accumulated smaller and more compact mitochondrial clusters [203].

The functional integrity of mitochondria is closely related to skin health. Mitochondria are important targets in anti-aging and repair, and skin care products based on mitochondrial repair can bring more benefits [204]. The main presence in the medical beauty industry is plant extracts or natural compounds that promote the generation of ATP or improve other mitochondrial functions. Artificial mitochondria transfer/transplant, an emerging technology, can alleviate skin aging by preventing the production of free radicals and providing an energetic boost. Although promising, this technology is still in the initial research stage [205, 206]. The development of an mtDNA-depleter-reporter mouse model further facilitated the exploration of potential drugs to prevent and slow skin aging and hair loss. The ongoing research includes transplanting young mitochondria to rejuvenate aging skin and hair [207]. An early cellular experimental study found that transferring mitochondria from homologous sources can repair damage caused by ultraviolet radiation and restore the mass and metabolic activity of mitochondria in cells [208]. In order to verify the efficacy of Pep-1 mediated mitochondrial transplantation on hair aging, Wu Hanchiang et al. [209] found that mitochondrial transplantation increased subcutaneous fat thickness stimulated hair regrowth and produced more growing follicles rich in dermal collagen. Mitochondrial transplantation has great potential in repairing skin tissue damage and restoring age-related hair loss. The development of regenerative medicine centered on mitochondria has greatly promoted the pace of human exploration of anti-aging. However, using mitochondrial transplantation to combat aging is still in its early stages. In the future, it is necessary to strengthen standardized management and promote the transformation of engineered mitochondria in this field.

## 6. Challenges and perspectives

Research in the field of anti-aging therapies and interventions involves various aspects such as lifestyle, nutrition, drugs, and technology. A balanced diet, moderate exercise, and a positive mental attitude help slow cellular aging and maintain physical health. Alternatively, the use of skin care products such as anti-wrinkle agents, moisturizers, and sunscreens, anti-aging drugs like antioxidant and anti-inflammatory agents, growth hormones, or supplements like Coenzyme Q10, grape seed extract, soy isoflavones can, to some extent, slow down cellular aging and improve bodily functions [210, 211]. Furthermore, interventions in the cellular aging process can be achieved through stem cell therapy or methods like gene editing to repair DNA damage. Mitochondrial transplantation, as a novel therapeutic strategy, provides a new therapeutic approach for mitochondrial disorders and anti-aging research. Compared to other anti-aging therapies and interventions, mitochondrial transplantation has some unique advantages and characteristics. 1) Mitochondrial transplantation directly repairs the functional impairment of mitochondria within cells, effectively enhancing cellular energy production and metabolic function. 2) Mitochondrial transplantation can be customized based on an individual's mitochondrial function status, increasing the treatment's specificity and effectiveness. 3) Compared to some short-term anti-aging interventions, mitochondrial transplantation may have longer-lasting effects. However, mitochondrial transplantation research is still in the early stages, and many mechanisms remain unexplained. More clinical trials and scientific validation are required to confirm its long-term efficacy, safety, and therapeutic benefits.

### 6.1 Storage of Mitochondria

A major obstacle in mitochondrial transplantation is the storage of isolated mitochondria. Under normal conditions, isolated mitochondria can maintain activity by

storing on ice for 1-2 hours [92, 212]. In a medium containing low Ca^2+^, maintaining a temperature below 4°C can preserve the mitochondrial outer membrane potential. However, over time, storage on ice for more than a certain period of time may lead to damage to membrane viability, thereby losing the ability to produce ATP [213]. Therefore, the shorter separation time window limits the application scope of mitochondrial transplantation. Establishing a method that allows for long-term storage of mitochondria will further expand clinical applications. Dimethyl sulfoxide (DMSO), glycerol, or trehalose have been shown to have some prospects in preserving OXPHOS. However, cytochrome c retention decreased after thawing, which may lead to inflammation [213]. Moreover, both DMSO and glycerol exhibit cytotoxic at high concentrations, which limits the application of current refrigeration techniques in mitochondrial storage [214]. Therefore, it is significant to establish a method that allows for long-term storage of mitochondria with high biosafety in future research.

### 6.2 Safety of mitochondrial transplantation

Mitochondrial transplantation may trigger an immune response, leading to the immune system attacking cells or tissues. In addition, transplanted mitochondria may not be completely stable, resulting in genomic instability. Autologous transplantation can reduce inflammation and rejection reactions. Most clinical mitochondria originate from patient muscle tissue. Another potential source solution is to collect proliferating primary cells (such as fibroblasts/platelets). Allogeneic mitochondria can also be used for mitochondrial transplantation, but there are still certain problems, and the source of mitochondria remains the current core issue that needs to be resolved. The host cell cannot absorb the damaged mitochondrial membrane, and the freely floating mtDNA can trigger an immune response [214]. Therefore, adjusting isolation techniques to obtain high-quality active intact mitochondria may assist clinical applications. Compared to differential and density gradient centrifugation, it is preferable to rapidly separate and purify mitochondria through differential filtration [215].

### 6.3 Strategies for mitochondrial-transfer optimization

Mitochondria have a short survival period after leaving cells and cannot directly target the damaged cells. Intravenous injection, as the main approach of mitochondrial transplantation, shows poor efficiency. Mitochondria are widely spread and sparsely distributed in the body, with only 3% to 7% of mitochondria entering target cells [71, 216, 217]. In order to solve the problem of mitochondrial targeting, Professor Gao Jianqing's team from the School of Pharmacy of Zhejiang University constructed a mitochondrial-targeted delivery system based on stem cell vectors, achieving efficient mitochondrial delivery into target damaged cells [218]. The team of Professor Sun Aijun from Fudan University and Professor Chen Zhaoyang from Fujian Medical University effectively binds mitochondria with a peptide CSTSMLKAC (PEP) and TPP to form a PEP-TPP-mitochondrial compound [219]. The PEP in this compound has ischemia-sensing properties, promoting its translocation to ischemic myocardium. In addition, the targeted peptide PEP can be easily separated from the compound, enabling effective internalization of mitochondria. Targeting mitochondria and delivering them to particular tissues is complex. Future research should concentrate on creating carriers tailored for precise cell delivery to enhance targeted mitochondrial delivery.

### 6.4 Patient selection and the role of personalized medicine

Most mitochondrial transplant research currently focuses on basic experiments with limited application in clinical trials. Only 7 clinical trials have been registered, mainly focusing on cerebral ischemia, infertility, myocardial infarction, and dermatomyositis. Patient selection plays a crucial role in mitochondrial transplantation. The patient's condition, medical history, and individual differences significantly impact the effectiveness of mitochondrial transplantation, hence the need to select appropriate candidates. A study has found that children with severe multisystem diseases, such as mitochondrial diseases or severe systemic oxalosis, are no longer suitable candidates for liver transplantation [220]. The criteria for suitable candidates for mitochondrial transplantation are currently unclear and require consolidation through numerous clinical trials. Personalized medicine plays a vital role in mitochondrial transplantation. Depending on the patient's specific circumstances, adjustments in the types, dosages, and administration of medications may be necessary to maximize the effects of mitochondrial transplantation. However, this remains to be seen due to limitations in clinical trials. The current situation above reminds us that in future research, we must pay attention to the timing, donor selection, and optimal conditioning protocol. By considering these factors comprehensively, we can continuously optimize mitochondrial transplantation to maximize treatment efficacy, reduce adverse reactions in patients, and promote their recovery.

### 6.5 Ethical issues

Mitochondrial replacement therapy (MRT) brings hope for fertility among women with severe inherited mitochondrial diseases. The UK was the first country to legalize mitochondrial donations in October 2015. In 2016, the first three-parent baby was born in Mexico [221]. However, as a way of preventing severe maternal mitochondrial genetic diseases, the safety and effectiveness of MRT still need more scientific data support. At present, MRT is still in the early stage of clinical trial. It also has scientific and safety controversies, as well as a series of ethical and social issues, such as "three-parent babies" and genetic modification of the human reproductive system. Several jurisdictions are actively promoting MRT policies. However, due to multidimensional factors such as ethical culture, and political stance, different jurisdictions have different attitudes and regulatory measures, which pose significant challenges to the development of MRT and management in this research field. In subsequent research, MRT needs to address the following issues. Firstly, the purpose of the MRT application should be defined, whether it is only allowed for the prevention of severe mitochondrial genetic diseases, or also for improving fertility. Secondly, define the terms genetic modification, gene modification, and gene editing in legal norms while clarifying whether these concepts only apply to nDNA or include the replacement of mtDNA simultaneously. Thirdly, clear regulatory boundaries should be established based on the substantive differences between nDNA and mtDNA. Fourthly, clarify which MRT can be used, as spindle transfer, pronuclear transfer, and polar body transfer involve specific operating objects and methods, different development maturity, and significant differences in application security risks, thus resulting in varying ethical acceptance levels. Fifthly, clarify whether only male embryos treated with MRT can be transplanted to block the possibility of donor mtDNA passage. Sixly, how to limit the patient's indications, such as the severity of mitochondrial diseases, the exhaustion of treatment options, and other factors. Sevenly, propose regulation of mitochondrial donation. Eightly, appropriate trade-offs should be made between the scientific competitiveness of biotechnology, the needs of patients, and the potential ethical and social issues of technology. Only by fully drawing on the common issues mentioned above in the international community's regulation of MRT application can we standardize and promote the application of MRT, thereby enhancing people's well-being.

## 7. Conclusion

Mitochondrial dysfunction is a prominent characteristic of aging, and maintaining healthy mitochondria and metabolic homeostasis are key to health. Numerous basic studies have shown that mitochondrial transplantation significantly improves mitochondrial function. However, clinical research on mitochondrial transplantation is still in the early stages, with numerous unresolved issues. In order to accelerate clinical application, the mitochondrial transplantation process (such as mitochondrial source, analysis, delivery methods, etc.) needs to be optimized to obtain more stable and targeted mitochondria. We believe that in the near future, mitochondrial transplantation can be widely applied in clinical and research fields with standardized guidelines.
